# The associations between MDM4 gene polymorphisms and cancer risk

**DOI:** 10.18632/oncotarget.10877

**Published:** 2016-07-28

**Authors:** Ming-Jie Wang, Yong-Jun Luo, Zhi-Yong Shi, Xiao-Liang Xu, Guo-Liang Yao, Rui-Ping Liu, Hui Zhao

**Affiliations:** ^1^ Department of Orthopedics, Affiliated Hospital of Nanjing Medical University, Changzhou Second People's Hospital, Changzhou 213003, China; ^2^ Department of Orthopedics, The First Affiliated Hospital of Nanjing Medical University, Nanjing 210029, China; ^3^ Department of Neurosurgery, Beijing Tiantan Hospital, Capital Medical University, China National Clinical Research Center for Neurological Diseases, Beijing 100050, China; ^4^ Department of Liver Surgery of Jiangsu Province People's Hospital, The First Affiliated Hospital of Nanjing Medical University, Nanjing 210029, China; ^5^ Department of Thoracic Surgery, The First Affiliated Hospital of Nanjing Medical University, Nanjing 210029, China; ^6^ Department of General Surgery, Third Affiliated Hospital of Nantong University, Wuxi, 214000, China

**Keywords:** MDM4, single nucleotide polymorphisms, meta-analysis, cancer

## Abstract

Considerable studies have investigated the associations between *MDM4* gene polymorphisms and cancer risk recently, but with contradictory results. The aim of this meta-analysis was to evaluate the associations between *MDM4* gene polymorphisms and cancer risk. Relevant studies were identified by a systematic search of PubMed, Embase, and CNKI databases. Crude odds ratios (ORs) and 95% confidence intervals (CIs) were used to describe the strength of the associations. Fifty-six studies published in 11 publications involving 18,910 cases and 51,609 controls were included in this meta-analysis. Five *MDM4* gene polymorphisms were evaluated: rs4245739, rs1563828, rs11801299, rs10900598, and rs1380576. Our analyses suggested that the rs4245739 polymorphism was significantly associated with overall cancer risk. Furthermore, stratification analyses of ethnicity indicated that rs4245739 decreased the risk of cancer among the Asian population, and stratification analyses of smoking status indicated that rs4245739 decreased the risk of cancer among nonsmokers. However, stratification analyses of cancer type and sex suggested that rs4245739 was not related to cancer risk. There were no associations of rs1563828, rs11801299, rs10900598, or rs1380576 with overall cancer risk. In conclusion, our analyses indicated that rs4245739 polymorphism in the *MDM4* gene may play an important role in the etiology of cancer.

## INTRODUCTION

Cancer is a worldwide health problem and the second leading cause of morbidity and mortality in human diseases [1]. Cancer metastasis to other parts of the body is the major cause of death [2]. Chen et al. reported that approximately 4,292,000 new cancer cases and 2,814,000 cancer deaths would occur in China in 2015 [3]. It is obvious that cancer is a major threat to human health. However, the exact mechanism of carcinogenesis is still poorly understood. An increasing number of studies have reported that cancer is a complex disease, resulting from environmental and genetic factors and their interactions [4, 5]. In addition, genetic factors play important roles in the pathogenesis of cancer, and many genes have been identified as cancer-susceptible genes [6].

The tumor suppressor p53 plays an important role in many physiological processes, such as maintenance of genomic stability and control of cell growth and apoptosis [7, 8]. Inactivation of the p53 tumor suppressor is critical for tumorigenesis. The activity of p53 is strictly regulated, predominantly by MDM2 and its homolog MDM4 [9]. MDM4 shares an N-terminal p53-binding domain with MDM2 and can inhibit p53 activity during various malignancies [10–12]. Overexpressed MDM4 in human tumors may contribute to reduced p53 activity and tumorigenesis [13]. Many studies [14, 15] have demonstrated that mouse embryos lacking MDM4 die during embryogenesis. Migliorini et al. found MDM4 regulates p53-induced growth arrest and neuronal cell death during early embryonic mouse development [14]. Furthermore, transgenic mice overexpressing MDM4 showed spontaneous carcinogenesis and accelerated tumorigenesis [16]. Additionally, MDM4 is reported to be highly expressed in a significant percentage of human cancers, including 65% of retinoblastomas, 80% of adult pre-B lymphoblastic leukemia, 39% of head and neck squamous carcinomas, 19% of colon cancers, 19% of breast cancers, and 18% of lung cancers [17–20]. All above studies indicated that *MDM4* gene may be significantly associated with cancer susceptibility.

Recently, many studies [13, 21–30] have described the associations between *MDM4* gene polymorphisms and risks of various cancers, including colon cancer, lung cancer, breast cancer, gastric cancer, squamous cell carcinoma of the head and neck, non-Hodgkin lymphoma (NHL), and esophageal squamous cell carcinoma (ESCC). However, the results of these studies were conflicting and inconclusive. The clinical heterogeneity, different ethnic populations, and small sample sizes of previous studies may have contributed to these disparities. To overcome these limitations, we performed a meta-analysis of the contradictory results from these relevant studies to clarify the possible associations between *MDM4* gene polymorphisms and cancer risk.

## RESULTS

### Characteristics of the included publications

**Figure 1:**
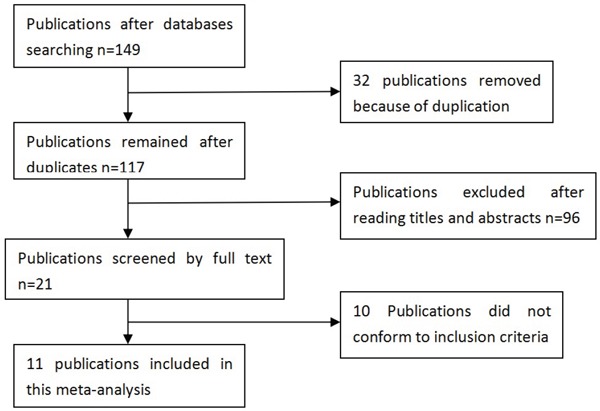
Selection for eligible publications included in this meta-analysis

A total of 149 publications were identified after our initial search. After removing duplicates and screening the titles and abstracts, 128 publications were removed. Finally, 21 publications were selected for further full text review. The following publications were excluded: four [31–34] in which the experimental designs were not case control studies; two publications [35, 36] that did not describe *MDM4* polymorphisms (rs4245739, rs1563828, rs11801299, rs10900598, and rs1380576) and cancer risk, three [37–39] that not provide detailed genotyping data, and two [28, 40] that might have described partially overlapping populations, so we included the study [28] with the larger sample size. We finally identified 11 eligible publications [13, 21–30] including 56 studies (18,910 cases and 51,609 controls) in this meta-analysis. Selection for eligible publications included in this meta-analysis was presented in Figure 1. The characteristics of these included studies are summarized in Table 1. These publications were published from 2011 to 2015. Five single nucleotide polymorphisms (SNPs) (rs4245739, rs1563828, rs11801299, rs10900598 and rs1380576) of *MDM4* gene were investigated. Genotype distributions of the controls about rs1380576 in one study [26] did not conform to HWE (*P* < 0.001). The NOS scores of all included studies ranged from 5 to 7 stars, suggesting that they were studies of high methodological quality. Four papers [13, 22, 27, 28] were carried out in Caucasian populations, and seven [21, 23–26, 29, 30] in Asian populations. The research [27] conducted by Garcia-Closaset et al. consisted of three genome-wide association studies (GWASs) involving 40 studies among Caucasian populations.

**Table 1:** Characteristics of included studies

Author and year	Country	Ethnicity	Case	Control					Cancer type	HWE	NOS
**rs4245739**			AA	AC	CC	AA	AC	CC			
Gansmo2015	Norway	Caucasian	823	600	108	2042	1439	266	Colon cancer	0.566	6
Gansmo2015	Norway	Caucasian	715	515	101	2042	1439	266	Lung cancer	0.566	6
Gansmo2015	Norway	Caucasian	1412	927	161	2042	1439	266	Prostate cancer	0.566	6
Gansmo2015	Norway	Caucasian	966	643	108	2042	1439	266	Breast cancer	0.566	6
Gao2015	China	Asian	297	22	1	548	90	2	SCLC	0.399	5
Gao2015	China	Asian	183	17	0	321	77	2	SCLC	0.248	7
Fan2014	China	Asian	187	13	0	346	53	1	NHL	0.487	6
Liu2013	China	Asian	733	67	0	686	111	3	Breast cancer	0.505	6
Liu2013	China	Asian	278	22	0	501	96	3	Breast cancer	0.484	6
Zhou2013	China	Asian	501	37	2	478	70	2	ESCC	0.740	6
Zhou2013	China	Asian	529	56	3	510	88	2	ESCC	0.379	6
Garcia-Closas2013	Mixed	Caucasian	3318	2637	557	22825	15798	2828	Breast cancer	0.183	5
**rs1563828**			CC	CT	TT	CC	CT	TT			
Zhang2012	China	Asian	98	91	21	90	88	22	NPC	0.944	7
Song2012	China	Asian	53	57	14	44	43	14	Breast cancer	0.506	5
**rs11801299**			GG	AG	AA	GG	AG	AA			
Wang2012	America	Caucasian	195	Na	Na	201	Na	Na	Oral cancer	Na	6
Yu2011	America	Caucasian	684	351	40	665	376	38	SCCHN	0.086	6
**rs10900598**			GG	GT	TT	GG	GT	TT			
Wang2012	America	Caucasian	107	Na	Na	94	Na	Na	Oral cancer	Na	6
Yu2011	America	Caucasian	307	545	223	296	552	231	SCCHN	0.377	6
**rs1380576**			CC	CG	GG	CC	CG	GG			
Wu2015	China	Asian	188	281	173	212	290	218	Gastric cancer	<0.001	5
Wang2012	America	Caucasian	141	Na	Na	149	Na	Na	Oral cancer	Na	6
Yu2011	America	Caucasian	487	477	111	518	455	106	SCCHN	0.677	6

HWE, Hardy–Weinberg equilibrium; NOS, Newcastle–Ottawa scale; NPC, Nasopharyngeal carcinoma; SCCHN, squamous cell carcinoma of the head and neck; SCLC, Small Cell Lung Cancer; NHL, Non-Hodgkin lymphoma; ESCC, esophageal squamous cell carcinoma; Na, not available.

### Meta-analysis of rs4245739

**Figure 2:**
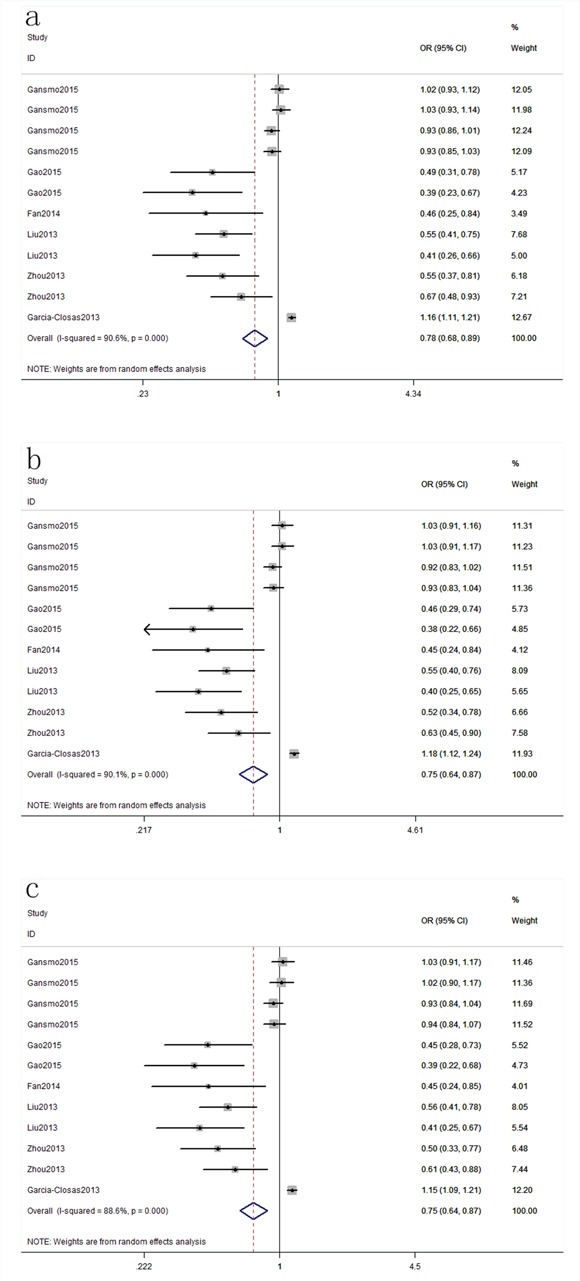
Forest plot shows odds ratio for the associations between rs4245739 and cancer risk (a: C vs. A; b: CC+AC vs. AA; c: AC vs. AA)

**Figure 3:**
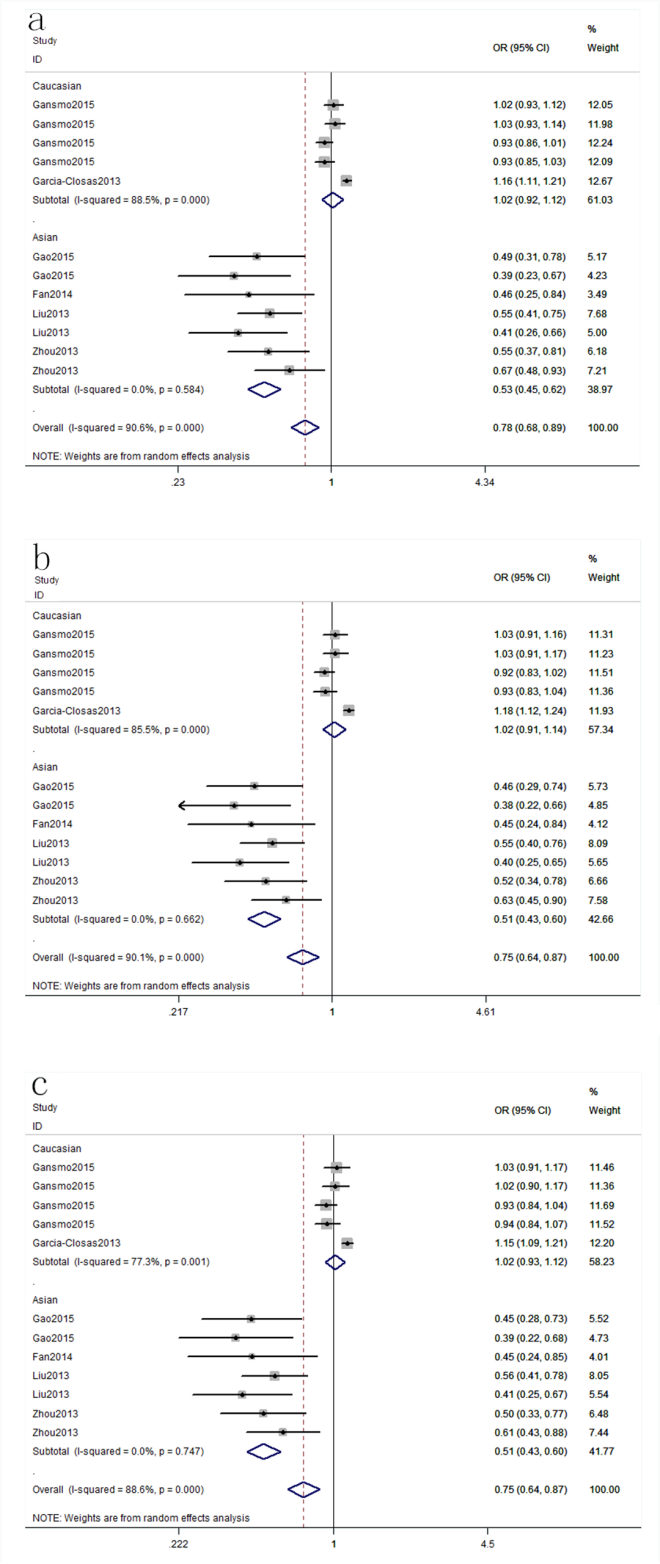
Stratification analyses of ethnicity between rs4245739 and cancer risk (a: C vs. A; b: CC+AC vs. AA; c: AC vs. AA)

**Figure 4:**
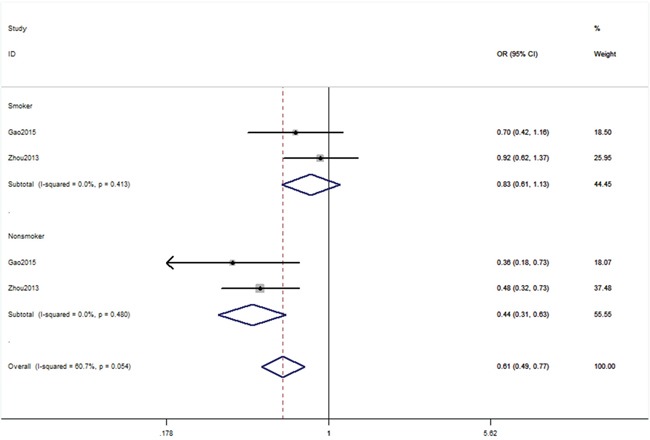
Stratification analyses of smoke status between rs4245739 and cancer risk (CC+AC vs. AA)

Six publications [13, 21, 24, 25, 27, 29] including 51 studies with 16,539 cases and 49,188 controls examined rs4245739 polymorphism. As shown in Table 2, rs4245739 polymorphism was significantly associated with a decreased risk of overall cancer risk in three models (C vs. A: OR, 0.78; 95% CI, 0.68–0.89, *P* < 0.001; CC+AC vs. AA: OR, 0.75; 95% CI, 0.64–0.87, *P* < 0.001; AC vs. AA: OR, 0.75; 95% CI, 0.64–0.87, *P* < 0.001, Figure 2). Stratification analyses were conducted according to ethnicity, cancer type, sex and smoking status. Our data indicated that rs4245739 polymorphism was also significantly associated with a decreased risk of cancer among Asian populations in three models (C vs. A: OR, 0.53; 95% CI, 0.45–0.62, *P* < 0.001; CC+AC vs. AA: OR, 0.51; 95% CI, 0.43–0.60, *P* < 0.001; AC vs. AA: OR, 0.51; 95% CI, 0.43–0.60, *P* < 0.001, Figure 3). Stratification analyses of smoking status indicated rs4245739 decreased the risk of nonsmokers (CC+AC vs. AA: OR, 0.44; 95% CI, 0.31–0.63, *P* < 0.001, Figure 4). However, stratification analyses of cancer type suggested rs4245739 was not related with the risks of lung cancer, breast cancer, gastrointestinal cancer and other cancers. Stratification analyses of sex also indicated this SNP was not associated with cancer risk. All included studies conform to HWE, indicating their controls subjects were representative of the general population.

**Table 2:** Meta-analysis of associations between the rs4245739 polymorphism and cancer risk

Comparison	Overall and Stratification analyses	Studies	OR (95% CI)	*P*-value	Random/Fixed effect model	*P* for heterogeneity	I^2^ (%)
C vs. A	Overall	51	**0.78(0.68,0.89)**	<0.001	Random	<0.001	90.6
	Caucasian	44	1.02(0.92,1.12)	0.301	Random	<0.001	88.5
	Asian	7	**0.53 (0.45,0.62)**	<0.001	Random	0.584	0.0
	Gastrointestinal cancer	3	0.74(0.49,1.12)	0.157	Random	0.001	85.6
	Lung cancer	3	0.61(0.31,1.19)	0.148	Random	<0.001	90.4
	Breast cancer	43	0.78 (0.59,1.02)	0.070	Random	<0.001	94.4
	Other cancers	2	0.70(0.35,1.39)	0.307	Random	0.024	80.5
CC vs. AA+AC	Overall	47	1.04(0.88,1.21)	0.666	Random	0.016	59.5
	Caucasian	44	1.03 (0.86,1.23)	0.748	Random	0.584	0.0
	Asian	3	1.20(0.38,3.84)	0.754	Random	0.032	78.1
	Gastrointestinal cancer	3	1.00 (0.80,1.26)	0.996	Random	0.895	0.0
	Lung cancer	2	1.07(0.85,1.36)	0.556	Random	0.953	0.0
	Breast cancer	41	1.08(0.75,1.55)	0.696	Random	0.003	88.4
	Other cancers	1	0.90 (0.74,1.10)	0.312	Na	Na	Na
CC+AC vs. AA	Overall	51	**0.75(0.64,0.87)**	<0.001	Random	<0.001	90.1
	Caucasian	44	1.02(0.91,1.14)	0.740	Random	<0.001	85.5
	Asian	7	**0.51(0.43,0.60)**	<0.001	Random	0.662	0.0
	Gastrointestinal cancer	3	0.72 (0.45,1.14)	0.157	Random	<0.001	87.0
	Lung cancer	3	0.59(0.29,1.20)	0.142	Random	<0.001	90.5
	Breast cancer	43	0.76(0.56,1.04)	0.089	Random	<0.001	94.0
	Other cancers	2	0.69 (0.34,1.39)	0.294	Random	0.025	80.0
	Male	5	0.72 (0.49,1.07)	0.107	Random	0.008	70.8
	Female	5	0.62 (0.32,1.19)	0.150	Random	<0.001	83.4
	Smoker	4	0.83 (0.61,1.13)	0.237	Fixed	0.413	0.0
	Nonsmoker	4	**0.44 (0.31,0.63)**	<0.001	Fixed	0.480	0.0
CC vs. AA	Overall	47	1.04(0.85,1.26)	0.360	Random	0.001	71.8
	Caucasian	44	1.03(0.83,1.28)	0.769	Random	<0.001	83.8
	Asian	3	1.13 (0.35,3.59)	0.840	Random	0.938	0.0
	Gastrointestinal cancer	3	1.01(0.80,1.28)	0.915	Random	<0.001	90.6
	Lung cancer	2	1.08(0.85,1.38)	0.522	Random	<0.001	88.5
	Breast cancer	41	1.09 (0.70,1.71)	0.698	Random	0.584	0.0
	Other cancers	1	0.88(0.71,1.08)	0.208	Random	0.032	78.1
AC vs. AA	Overall	51	**0.75 (0.64,0.87)**	<0.001	Random	<0.001	88.6
	Caucasian	44	1.02 (0.93,1.12)	0.694	Random	0.001	77.3
	Asian	7	**0.51(0.43,0.60)**	<0.001	Random	0.747	0.0
	Gastrointestinal cancer	3	0.70(0.43,1.14)	0.157	Random	<0.001	87.7
	Lung cancer	3	0.58(0.29,1.19)	0.136	Random	<0.001	90.0
	Breast cancer	43	0.78(0.58,1.04)	0.091	Random	<0.001	92.6
	Other cancers	2	0.70(0.35,1.39)	0.307	Random	0.028	79.3

*Bold values are statistically significant (*P* < 0.05). OR, odds ratio; 95% CI, 95% confidence interval; Na, not available.

### Meta-analysis of rs1563828, rs11801299, rs10900598 and rs1380576

Two publications [23, 30] with 334 cases and 301 controls examined rs1563828 polymorphism; two publications [22, 28] with 1,395 cases and 1,400 controls studied rs11801299 polymorphism; two publications [22, 28] with 1,395 cases and 1,400 controls studied rs10900598 polymorphism; three publications [22, 26, 28] with 2,037 cases and 2,120 controls studied rs1380576 polymorphism. With regard to rs11801299, rs10900598 and rs1380576, we could only calculate the data in dominant model as they did not provide enough data. As shown in Table 3, we found these four SNPs were not associated with cancer risk. For these four SNPs, we did not perform stratification analyses due to limited data. Genotype distributions of the controls about rs1380576 in one study [26] did not conform to HWE (*P* < 0.001). By exclusion of this study, the pooled estimates of the remaining studies showed that rs1380576 polymorphism was also not associated with cancer risk (CG+GG vs. CC: OR, 1.11; 95% CI, 0.96–1.29, *P* = 0.164), suggesting that the results of this SNP was stable.

**Table 3:** Meta-analysis of associations between rs1563828, rs11801299, rs10900598 and rs1380576 polymorphisms and cancer risk

Comparison	OR(95%CI)	*P*-value	Random/Fixed effect model	*P* for heterogeneity	I^2^ (%)
**rs1563828**					
T vs. C	0.95(0.75,1.20)	0.658	Fixed	0.928	0.0%
TT+CT vs. CC	0.96(0.70,1.32)	0.814	Fixed	0.744	0.0%
TT vs. CC+CT	0.86(0.52,1.40)	0.536	Fixed	0.804	0.0%
CT vs. CC	1.00(0.72,1.39)	0.997	Fixed	0.678	0.0%
TT vs. CC	0.86(0.51,1.45)	0.566	Fixed	0.921	0.0%
**rs11801299**					
AG+AA vs. GG	0.95(0.82,1.11)	0.529	Fixed	0.399	0.0%
**rs10900598**					
GT+TT vs. GG	0.91(0.78,1.08)	0.288	Fixed	0.483	0.0%
**rs1380576**					
CG+GG vs. CC	1.08(0.95,1.22)	0.227	Fixed	0.785	0.0%

*Bold values are statistically significant (*P* < 0.05).

### Sensitivity analysis and publication bias

**Figure 5:**
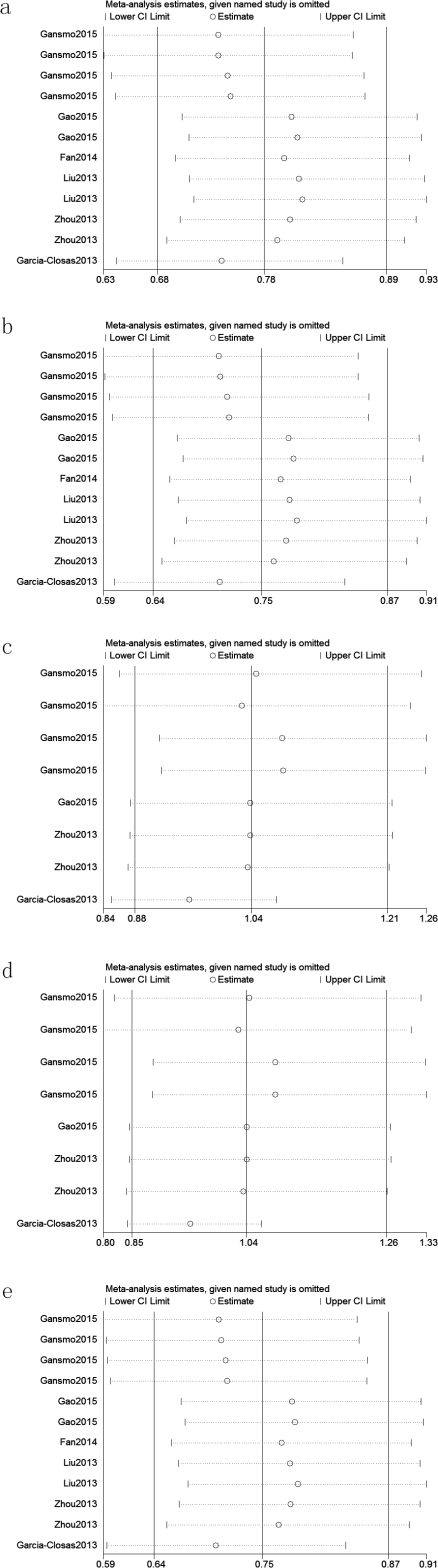
Sensitivity analyses between rs4245739 and cancer risk in five models (a: allele model; b: dominant model; c: recessive model; d: homozygous model; e: heterozygous model)

We assessed sensitivity by omitting each study once at a time in every genetic model for rs4245739. The pooled ORs for the effects of rs4245739 on the risk for cancer risk indicated that our data were stable and trustworthy about this SNP (Figure 5). We did not perform sensitivity analyses about rs1563828, rs11801299, rs10900598 and rs1380576 due to limited data. Both Egger's and Begg's tests were used to evaluated the publication bias of this meta-analysis. Our data revealed that there was obvious publication bias in three models for rs4245739 (C vs. A, *P*_begg_ = 0.075 and *P*_egger_ < 0.001; CC+AC vs. AA, *P*_begg_ = 0.055 and *P*_egger_< 0.001; AC vs. AA, *P*_begg_ = 0.040 and *P*_egger_ < 0.001). Due to limited studies, we did not conduct Egger's and Begg's tests about rs1563828, rs11801299, rs10900598 and rs1380576.

## DISCUSSION

To the best of our knowledge, this is the first meta-analysis emphasizing the associations between *MDM4* gene polymorphisms and cancer risk. Our data indicated that the rs4245739 polymorphism was significantly associated with a decreased risk of cancer overall. In addition, stratification analyses of ethnicity indicated that rs4245739 decreased the risk of cancer in the Asian population, and stratification analyses of smoking status indicated that rs4245739 decreased the risk of cancer among nonsmokers. However, stratification analyses of cancer type suggested rs4245739 was not related to the risks of lung cancer, breast cancer, gastrointestinal cancer, and other cancers. Stratification analyses of sex also indicated this SNP was not associated with cancer risk in male or female populations. With regard to the remaining four SNPs, no associations were found between rs1563828, rs11801299, rs10900598, or rs1380576and overall cancer risk.

One of most important tumor suppressors in human cells is p53. MDM2, a key regulator of the p53 tumor suppressor signaling pathway, can induce degradation of p53 through the ubiquitin-proteasome pathway [41]. MDM4 is structurally homologous to MDM2, and MDM4 can cooperate with MDM2 to inhibit p53 activities [10]. Furthermore, MDM4 can interact with MDM2 via the Really Interesting New Gene finger domain that inhibits the degradation of MDM2 protein [12, 42]. In light of these findings, we hypothesize that MDM4 may play pivotal roles in the pathogenesis of cancer, and that *MDM4* is a candidate susceptibility gene for cancer. Currently, many studies [13, 21–30] have investigated the associations between *MDM4* gene polymorphisms and cancer risk. However, these studies showed inconsistent results, because they had inadequate statistical power due to relatively small samples.

To provide a comprehensive and reliable conclusion, we conducted a meta-analysis to assess the associations between *MDM4* gene polymorphisms and cancer risk. Our data indicated that the rs4245739 polymorphism was significantly associated with a decreased risk of overall cancer. Six publications [13, 21, 24, 25, 27, 29] including 51 studies involving 16,539 cases and 49, 188 controls investigated this SNP. Among these included publications, four [21, 24, 25, 29] from China assessed this variant in ESCC, small cell lung cancer (SCLC), and NHL and breast cancer and concluded that rs4245739 was associated with a reduced risk of cancer. Studies from Norway [13] suggested that rs4245739 is associated with a reduced risk of breast cancer but is not associated with lung cancer, colon cancer, or prostate cancer. Studies by Garcia-Closaset et al. [27], consisting of three GWAS studies among Caucasian populations, indicated that rs4245739 increased the risk of breast cancer. As mentioned previously, we found that the associations between rs4245739 and breast cancer risk were inconsistent. Two studies [13, 24] reported that rs4245739 decreased the risk of breast cancer, while the study by Garcia-Closaset et al. [27] reported that it increased the risk of breast cancer. It is noteworthy that Garcia-Closaset et al. reported that rs4245739 increased the risk of estrogen receptor (ER)-negative but not ER-positive breast cancer, which was consistent with the conclusion of two other GWAS studies [37, 38]. There are important differences in genetic susceptibility with these two types (ER-negative and ER-positive) of breast cancer. Garcia-Closaset et al. reported that rs4245739 is located in an ER-negative-specific breast cancer risk locus. It is therefore reasonable to hypothesize that this SNP may specifically affect susceptibility to ER-negative breast cancer. Stratification analyses of cancer type in this meta-analysis concluded that rs4245739 is not associated with overall breast cancer. The reasons why the results of Garcia-Closaset et al. differed from the results of those two studies [13, 24] and this meta-analysis are unclear, but it may be partially explained by differences in the genetic susceptibility of different types of breast cancer. Due to limited data, we could not conduct stratification analyses of ER status. Larger studies are therefore needed to identify the possible association between rs4245739 and ER-negative and ER-positive breast cancer.

Stratification analyses of ethnicity suggested rs4245739 decreased the risk of cancer in Asian population. The genetic background of cancer may vary among different ethnicities. We did not find that rs4245739 was associated with the risk of cancer in Caucasians, although three GWAS studies [27, 37, 38] reported that this SNP increased the risk of ER-negative or triple negative breast cancer (defined by the absence of ER, progesterone receptor and human epidermal growth factor receptor-2) in the Caucasian population. In this meta-analysis, a large difference was found in the distribution of rs4245739 between Caucasians and Asians, with minor allele frequencies of 26.2% and 6.2%, respectively, and this was possibly affected by the power of the studies conducted in Asian populations and the final relationships between the rs4245739 polymorphism and cancer risks among different racial groups [13]. Furthermore, the etiology of ER-negative breast cancer is different from that of ER-positive breast cancer, including differences in genetic predisposition [43, 44]. Therefore, it is reasonable to assume that the genetic susceptibility of ER-negative or triple negative breast cancer is different than those of other breast cancer subtypes or overall breast cancer, which may explain why the results of these GWAS studies in Caucasians are different from the results of this meta-analysis in Caucasians. The reasons why rs4245739 decreased the risk of cancer among Asian population may be that the functional rs4245739 SNP A>C locating in the MDM43′-untranslated (3′-UTR) region creates a miR-191-5p or miR-887-3p targeting sites [23]. MiR-191-5p and miR-887-3p could bind to MDM43′-UTR with the rs4245739 C allele selectively [23]. These changes could result in decreased expression of oncogene MDM4, which could reduce the inhibition of p53 activities. Stratification analyses of smoking status indicated that rs4245739 decreased the risk of cancer among nonsmokers. Two studies [21, 29] investigating SCLC and ESCC were included in those stratification analyses. The studies also revealed significant multiple interactions between rs4245739 and smoking. Notably, SCLC and ESCC share similar environmental etiologies such as heavy smoking.

Regarding the remaining four SNPs, we failed to identify any associations between rs1563828, rs11801299, rs10900598, and rs1380576 and cancer risk. We cannot definitively conclude that these four SNPs are not associated with cancer risk, because this meta-analysis included only a few studies with limited sample sizes, and any associations between gene polymorphisms and disease are greatly affected by the number of participants. Given the limited sample size, the relationships between these four SNPs and cancer risk should be interpreted with caution. Genotype distributions of the controls in one study [26] on rs1380576 did not conform to HWE. After excluding this study, the conclusions of the remaining studies did not significantly change, suggesting that the result of rs1380576 was trustworthy.

Several potential limitations of this meta-analysis should be considered. First, our data indicated that publication bias existed with respect to studies regarding rs4245739, and potential language bias may have resulted from the inclusion of published studies in English or Chinese only. Second, the number of studies on rs1563828, rs11801299, rs10900598, and rs1380576 included in the meta-analysis was small, and the sample size was limited, which prevented further stratification analyses of other potential factors. Third, our results were based on unadjusted estimates for confounding factors, which might have affected the final results. Fourth, we could not assess potential gene-gene and gene-environment interactions because of the lack of original data. Fifth, this meta-analysis only included Asian and Caucasian populations; future studies on other ethnic groups are necessary because of ethnic differences in gene polymorphisms. Sixth, heterogeneity was considerable in this meta-analysis because the included studies involved different ethnicities and environments. Although sensitivity analyses indicated that our data were stable and trustworthy, we should interpret these data with caution.

In conclusion, this meta-analysis indicates that rs4245739 polymorphism of *MDM4* gene plays important roles in cancer pathogenesis, especially among Asian populations. Stratification analyses also indicate that rs4245739 decreases the risk of cancer among nonsmokers. However, the other four SNPs are not associated with cancer risk. Larger well-designed studies are necessary to validate these findings.

## MATERIALS AND METHODS

### Literature search

We systematically searched the PubMed, Embase, and China Knowledge Resource Integrated Database to identify studies through January 1, 2016. The following search terms were used: “cancer,” “carcinoma,” “neoplasm,” “tumor,” “MDM4,” “MDMX,” “HDMX,” “polymorphism,” “SNP” and “polymorphisms”. Two independent authors conducted the search. No language or other restrictions were placed on the search. Additional initially omitted studies have been identified by hand screening.

### Criteria of inclusion and exclusion

The included studies conformed to the following criteria: (1) studies that evaluated the associations between cancer risk and *MDM4* gene polymorphisms (at least one of the five polymorphisms), (2) studied on human beings, (3) study provided sufficient data to calculate the odds ratios (ORs) and 95% confidence intervals (CIs), and *P* value, and (4) case-control study. Exclusion criteria were: (1) duplication of previous publications; (2) case reports or review articles; (3) studies without detailed genotype data.

### Data extraction and quality assessment

Relevant information was carefully extracted from all eligible studies. The extracted information including: name of first author, publication year, country of origin, ethnicity, numbers of cases and controls, and cancer type. Two authors independently performed the extraction of data and assessed the study quality based on the Newcastle-Ottawa Scale (NOS) [45]. Total NOS scores ranged from 0 to 9. A score ranging 5 to 9 stars is considered to be a generally high methodological quality whereas a score ranging 0 to 4 is regarded as a relatively poor quality. All disagreements were discussed and resolved with consensus.

### Statistical analysis

All statistical analyses were performed using the Stata 11.0 software (StataCorp, College Station, TX, USA). The strength of associations between *MDM4* gene polymorphisms and cancer risk were estimated for each study by crude ORs and 95% CIs. Stratification analyses were carried out by ethnicity, cancer type, sex and smoking status. *P* < 0.05 was considered statistically significant. When a significant Q test (*P* < 0.1) or I^2^ < 50% indicated heterogeneity across studies, a fixed-effect model was used. Otherwise, the fixed-effects model was applied [46]. Pooled ORs were calculated for allele model, dominant model, recessive model, homozygous model, and heterozygous model. We performed sensitivity analyses by omitting each study in turn to determine the effect on the test of heterogeneity and evaluated the stability of the overall results. Hardy–Weinberg equilibrium (HWE) was assessed in the controls using Pearson's χ^2^ test. Potential publication bias was assessed by Begger's and Egger's linear regression test [47]; *P* < 0.05 was considered to indicate statistically significant.

## Abbreviations

SCCHN: squamous cell carcinoma of the head and neck
NHL: Non-Hodgkin lymphoma
ESCC: esophageal squamous cell carcinoma
SCLC: small cell lung cancer
CI: confidence interval
OR: odds ratio
NOS: Newcastle-Ottawa Scale
HWE: Hardy–Weinberg equilibrium
SNP: single nucleotide polymorphism
